# Motives for self-referral to the emergency department: a systematic review of the literature

**DOI:** 10.1186/s12913-016-1935-z

**Published:** 2016-12-09

**Authors:** Nicole Kraaijvanger, Henk van Leeuwen, Douwe Rijpsma, Michael Edwards

**Affiliations:** 1 Emergency Department, Rijnstate Hospital, Wagnerlaan 55, Arnhem, The Netherlands; 2 Department of Intensive Care / Internal Medicine, Rijnstate Hospital, Wagnerlaan 55, Arnhem, The Netherlands; 3 Department of Surgery, Radboud University Medical Centre, Geert Grooteplein-Zuid 10, Nijmegen, The Netherlands

**Keywords:** Self-referred patients, Emergency department, Systematic review

## Abstract

**Background:**

In several western countries patients’ use of Emergency Departments (EDs) is increasing. A substantial number of patients is self-referred, but does not need emergency care. In order to have more influence on unnecessary self-referral, it is essential to know why patients visit the ED without referral. The goal of this systematic review therefore is to explore what motivates self-referred patients in those countries to visit the ED.

**Methods:**

Recommendations from the PRISMA were used to search and analyze the literature. The following databases; PUBMED, MEDLINE, EMBASE, CINAHL and Cochrane Library, were systematically searched from inception up to the first of February 2015. The reference lists of the included articles were screened for additional relevant articles. All studies that reported on the motives of self-referred patients to visit an ED were selected. The reasons for self-referral were categorized into seven main themes: health concerns, expected investigations; convenience of the ED; lesser accessibility of primary care; no confidence in general practitioner/primary care; advice from others and financial considerations. A random-effects meta-analysis was performed.

**Results:**

Thirty publications were identified from the literature studied. The most reported themes for self-referral were ‘health concerns’ and ‘expected investigations’: 36% (95% Confidence Interval 23–50%) and 35% (95% CI 20-51%) respectively. Financial considerations most often played a role in the United States with a reported percentage of 33% versus 4% in other countries (*p* < 0.001).

**Conclusions:**

Worldwide, the most important reasons to self-refer to an ED are health concerns and expected investigations. Financial considerations mainly play a role in the United States.

## Background

The utilization of Emergency Departments (EDs) is increasing in several high-income countries [1, 2]. Inappropriate presentations to EDs are a burden for healthcare systems, contributing to excess diagnostics and treatment, overcrowding of EDs and longer waiting times; all are associated with increasing health care costs [3–5]. This is important, because worldwide health care expenditures as a share of gross domestic product are increasing over the last years [6]. In addition, using the ED for primary care problems reduces continuity of care for patients.

Several countries experience high percentages of self-referred ED-patients. In England, 62.8% of ED-patients is self-referred [1]. In the United States (USA), relatively few general practitioners (GPs) are available and patients often self-refer to EDs or other types of specialized care [7]. In the Netherlands, despite its strong primary care network, 30% of ED-patients is self-referred [8]. Within the category of self-referred patients is a substantial number of patients that could have been taken care of in primary care. In a previous study, our group found that between 41.2 to 51.9% of self-referred patients in a Dutch ED visited the ED inappropriately [9]. This is crucial, because strategies that aim to reduce ED utilization should target inappropriate self-referral.

In order to reduce inappropriate self-referral, it is essential to know why patients visit the ED directly. The goal of this systematic review is to explore what motivates self-referred patients worldwide to visit the ED directly.

## Methods

Recommendations from the Preferred Reporting Items in Systematic Reviews and Meta-Analysis (PRISMA) were followed [10].

### Search strategy and data sources

The following five databases: PUBMED, MEDLINE, EMBASE, CINAHL and Cochrane Library, were systematically searched from inception up to the first of February 2015. Searches were conducted using a combination of the following search terms: emergency department, self-referred, referral, walk-in, motives and reasons with appropriate wildcards and variations in spelling. The search in Pubmed was as follows: (“Emergency Service, Hospital” [Mesh] OR “emergency department” OR “emergency room” OR “emergency unit” OR “emergency service” OR “emergency ward”) AND (self-refer* OR refer* OR walk-in*) AND (motiv* OR reason*), no limits were used. A similar search was conducted for the other databases.

The reference lists of the included articles were screened for additional relevant articles.

### Inclusion criteria

Inclusion criteria were: study participants were self-referred patients in the ED (not referred by a GP and not brought in by ambulance), the study reported on reasons for patients to visit the ED without referral. All age groups and all disease categories were included. Different methods to study these motives were accepted. Only articles in English and Dutch language were included.

### Data extraction

**Fig. 1:**
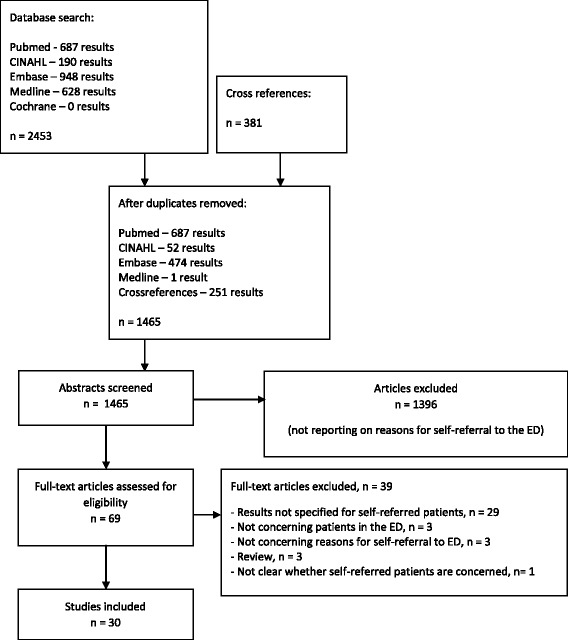
PRISMA flow diagram

Two authors (NK and HL) independently and in duplicate reviewed the titles and abstracts of retrieved publications and subsequently the full text was reviewed for possibly relevant articles. From the included articles, data on study purpose, design, setting, sample size, patient characteristics, study quality and country where the study was conducted was extracted. Disagreements were resolved by discussion until consensus was reached. The PRISMA flow diagram is shown in Fig. 1.

All different reasons for self-referral that were reported in the studies were listed. From these lists, seven themes for reasons for self-referral were identified by the study group (expert opinion) and consensus was reached within our group. Subsequently, the different reasons for self-referral that were found in the included articles were categorized into the seven themes. The themes were: health concerns; expecting investigations; convenience of the ED; lesser accessibility of primary care; no confidence in GP/primary care; advice from others; financial considerations (Appendix 1, 2).

### Statistical analysis

A random-effects meta-analysis was used in which all eligible studies were included. The meta-analysis was performed using the inverse variance method, with an empirical Bayes estimator for the heterogeneity parameter tau^2^, a Hartung-Knapp adjustment, and an arcsine transformation of proportions. Results of the primary studies were reported with Clopper-Pearson exact confidence intervals. The software R, version 4.1-0, package meta, from Guido Schwarzer (2015) was used [11].

In order to investigate whether the differences in reasons for self-referral could be explained by different healthcare systems or different study methods, the following subgroup analyses were performed: reporting on a specific condition; continent; including multiple choice questions; possibility to select multiple answers with multiple choice questions; including a Likert Scale; the year in which studies were published in; inclusion of only patients with non-urgent medical problems; and included age group (children, adults, all ages).

## Results

### Selected studies

Thirty studies were included, reporting motives for self-referral of 16450 patients [3, 5, 11–38]. The number of included patients differed considerably between the selected studies. Patient characteristics and study methodology were heterogeneous. Sixteen studies only included patients with non-urgent problems. [12, 14, 17, 19, 22, 24, 25, 27, 29–31, 34, 35, 37–39] Sixteen studies made use of questionnaires [3, 5, 12, 13, 16–19, 27, 31–33, 36–39], often with multiple choice questions [3, 5, 12, 13, 16, 19, 22, 27, 33, 37, 39] Three studies performed interviews with qualitative methodologies [29, 30, 34]. Others performed interviews without qualitative methods, sometimes by telephone, or by letting the treating physician or triage nurse ask one open question [14, 15, 20–26, 28, 31, 32, 35].

Most of the studies were performed in Europe and of the 19 European studies [3, 5, 11–27], 12 studies were performed in the United Kingdom (UK) [12, 14, 16, 18, 19, 21–26, 28]. The remaining studies were performed in the Netherlands [3, 5, 13, 17, 20], Ireland [15], Denmark [27], USA [29–34], Australia [37, 38], Hong Kong [35], Kuwait [36], and Israel [39] (Table 1).

**Table 1:** Selected studies, investigating motives for self-referral to the ED

	Article	Country, year of publication	Method	Number of patients	Inclusion/exclusion
Europe					
1	Mestitz [28]	UK 1957	Questions asked by casualty medical officer	975 (770 SRPs)	Only adults?
2	Wilkinson et al. [24]	UK 1977	Interviews, using questionnaires	546 (213 SRPs)	All ages Non-urgent
3	Myers et al. [26]	UK 1982	Question asked	150	Only adults?
4	Singh [21]	UK 1988	Interviews, using semi-structured questionnaire	217	All ages
5	O’Halloran et al. [16]	UK 1989	Postal questionnaires	145 (124 SRPs)	Age: 18 months to 16 years. Acute asthma
6	Stewart et al. [18]	UK 1989	Questionnaires	853 (585 SRPs)	Children
7	Thomson et al. [19]	UK 1995	Questionnaires	245 (147 SRPs)	Only adults? Non-urgent
8	Ward et al. [25]	UK 1996	Question asked by treating physician	970 (339 patients answered question)	All ages Non-urgent
9	Laffoy et al. [15]	Ireland 1997	Questionnaires, interviewer-administered	557 (395 SRPs)	All ages
10	Shipman et al. [23]	UK 1997	Telephone interviews, semi-structured	82	All ages
11	Rieffe et al. [17]	Netherlands 1999	Questionnaires, Likert scale	430	Only adults? Non-urgent
12	Jaarsma-van Leeuwen et al. [5]	Netherlands 2000	Questionnaires	1068	All ages Only surgical patients
13	Rajpar et al. [22]	UK 2000	Interviews, using semi-structured questionnaire	54	All ages Non-urgent
14	Coleman et al. [12]	UK 2001	Questionnaires	255	Adults Non-urgent
15	Norredam et al. [27]	Denmark 2007	Questionnaire	3426 (2746 SRPs)	Age > 14 years Non-urgent
16	Moll van Charante et al. [3]	Netherlands 2008	Postal questionnaires	808 (224 SRPs)	All ages
17	Mc Guigan et al. [14]	UK 2010	Interviews by telephone, semi-structured	196	Age > 16 years Non-urgent
18	van der Linden et al. [20]	Netherlands 2014	Open question by triage nurse	3028 (1751 patients answered question)	All ages
19	de Valk et al. [13]	Netherlands 2014	Questionnaires	436	Age > 18 years
North America					
20	Hunt et al. [33]	USA 1996	Questionnaires	1538	All ages
21	Koziol-McLain et al. [34]	USA 2000	Interviews, qualitative methodology	30	Age > 18 years Non-urgent
22	Northington et al. [31]	USA 2004	Questionnaire + brief interview	279	Age > 18 years Non-urgent
23	Howard et al. [30]	USA 2005	Interviews, qualitative methodology	31	Age 18–50 years Non-urgent
24	Ragin et al. [32]	USA 2005	Interviews + questionnaires with Likert scale	1536	Age > 18 years
25	Grant et al. [29]	USA 2010	Interviews, qualitative methodology	112	Children Non-urgent
Asia					
26	Shah et al. [36]	Kuwait 1996	Questionnaires, open ended question	1146	Only adults?
27	Lee et al. [35]	Hong Kong 2000	Telephone interviews, using questionnaires	2410 (726 patients answered question)	All ages Non-urgent
Australia					
28	Masso et al. [38]	Australia 2007	Questionnaire, Likert scale	397	All ages Non-urgent
29	Siminski et al. [37]	Australia 2008	Questionnaires	400	All ages Non-urgent
Other					
30	Rassin et al. [39]	Israel 2005	Questionnaire	73	Age > 18 years Non-urgent

*SRPs* self-referred patients

### Reasons for self-referral

Various motives for self-referral were found, with overlapping motives between studies. Percentages of the reasons reported by different studies were divergent. The reasons for self-referral were categorized into seven themes: health concerns; expecting investigations; convenience of the ED; lesser accessibility of primary care; no confidence in GP/primary care; advice from others; financial considerations. The different themes with examples are shown in Table 2.

**Table 2:** Examples of the seven different themes

Theme	Examples cited in articles
Health concerns	- Perceived severity of problem - Seeking assurance - Patient perceived the complaint was urgent
Expecting investigations	- Further research (eg X-rays) was necessary - Perceived facilities and investigations better at A&E - See doctor and have tests/x-rays done in same place
Advice of others	- On the advice of others - Sent by someone (usually employer) - They were referred by the staff (not the doctor) in PCP’s offices to be evaluated in the ED
Convenience of ED	- Patient could get help earlier at the ED - The ED was nearby - Convenience of access
Accessibility of GP	- Patient could not reach the GP/GP-cooperative - Unavailability of GP - Too long wait for family doctor
Financial considerations	- Payment flexibility - Affordability - Low cost
No confidence in GP	- Patient had no faith/trust in the GP - Previous negative experience with the GP/GP-cooperative - Dissatisfied with GP

To find the most common reasons for self-referral, a meta-analysis was performed; the results are shown in Table 3.

**Table 3:** Results of the meta-analysis, showing per theme the number of patients and studies and the percentage of patients indicating this theme as reason for their visit to the ED

Theme	Number of studies	Number of patients in these studies	% patients	95% CI (%)	I^2^ (%)	95% PI (%)
Health concerns	22	5564	36	23 – 50	99.7	0 – 94
Expecting investigations (radiological/blood tests)	10	1316	35	20 – 51	98.1	1 – 85
Advice of others	9	346	19	6 – 37	97.9	0 – 80
Convenience of ED	21	2939	18	11 – 26	99.5	0 – 62
Accessibility of GP	17	1744	13	9 – 18	92.4	0 – 36
Financial considerations	6	575	11	1 – 30	99.1	0 – 74
No confidence in GP	5	93	5	1 – 15	90.9	0 – 40

*CI* Confidence Interval

I^2^: the percentage of the total variation across studies due to heterogeneity; it takes values from 0-100% with the value of 0% indicating no observed heterogeneity

*PI* Prediction interval: expected 95% range of outcomes, where the results of a new study would fall within

Health concerns were reported by 36% of the patients. This theme was reported by studies from all continents, and in studies including patients with urgent and non-urgent conditions [3, 12–18, 20–22, 24, 25, 27, 29, 31–33, 35–39].

Several factors that were related to the high variability in the reported percentages of health concerns were found. The two studies performed in Australia [37, 38] found the highest percentage of patients indicating health concerns as a reason for self-referral: 74% (95% CI 4-100%), versus 48% (95% CI 2–98%) in the USA [31–33], 25% (95% CI 13 – 41%) in Europe [3, 12–18, 20–22, 24, 25, 27] and 24% (95% CI 0 – 100%) in Asia [35, 36] (*p* = 0.0003).

Health concerns were reported in 14% (95% CI 0–52%) in studies including only children [16, 18], versus 47% (95% CI 14–81%) in studies including only adults [12–14, 27, 31, 32, 36, 39] and 33% (95% CI 20–48%) in studies including patients of all ages [3, 15, 20–22, 24, 25, 33, 35, 37, 38] (*p* = 0.0014).

Both the year in which a study was published and the use of a Likert scale had a small influence on the heterogeneity regarding health concerns; reflected by an I^2^ remaining higher than 97%.

Thirty-five percent of the self-referred patients visited the ED because they expected to need laboratory or radiological investigations. The studies reporting on this reason for self-referral were all conducted in either Europe [3, 5, 12, 13, 15, 21–23, 26, 28] or Australia [37, 38].

Studies performed in Australia reported that 63% (95% CI 0 – 100%) of the included patients indicated this theme, compared to 28% (95% CI 16–44%) in studies from Europe (*p* = 0.01). Other subgroup analyses did not show significant associations.

The theme ‘advice from others’ was reported by 19% (PI 0-80%) of self-referred patients. In studies including only non-urgent patients [12, 14, 24, 25, 39] this theme was reported by 32% (95% CI 7 – 65%), versus 6% (95% CI 2 – 11%) in studies also including urgent patients [13, 16, 21, 26].

The year in which studies were performed also had an influence on the heterogeneity regarding the theme ‘advice from others’, which is probably explained by the fact that all studies published between 2000 and 2010 reporting on ‘advice from others’, included only non-urgent patients [12, 14, 39].

‘Convenience of the ED’ was reported by 18% (PI 0-62%) of self-referred patients. There were no subgroups with a significant relation to this theme.

The theme ‘accessibility GP’ was indicated by 13% (PI 0-36%) of self-referred patients. Multiple studies found patients claiming their GP is not available or not having a personal GP [3, 5, 12, 13, 17, 20–26, 29, 32, 35]. Several studies found patients declaring they did not think of their GP, were not aware of other services, such as a walk-in clinic or GP-cooperative, or did not know the location of an alternative service [5, 11, 12, 21, 22, 32]. Also within this theme, several studies found that patients turned to the ED, because they felt they had to wait too long for an appointment with their GP [5, 17, 23, 25, 28, 32] No statistically significant differences were found in subgroup analyses.

**Fig. 2:**
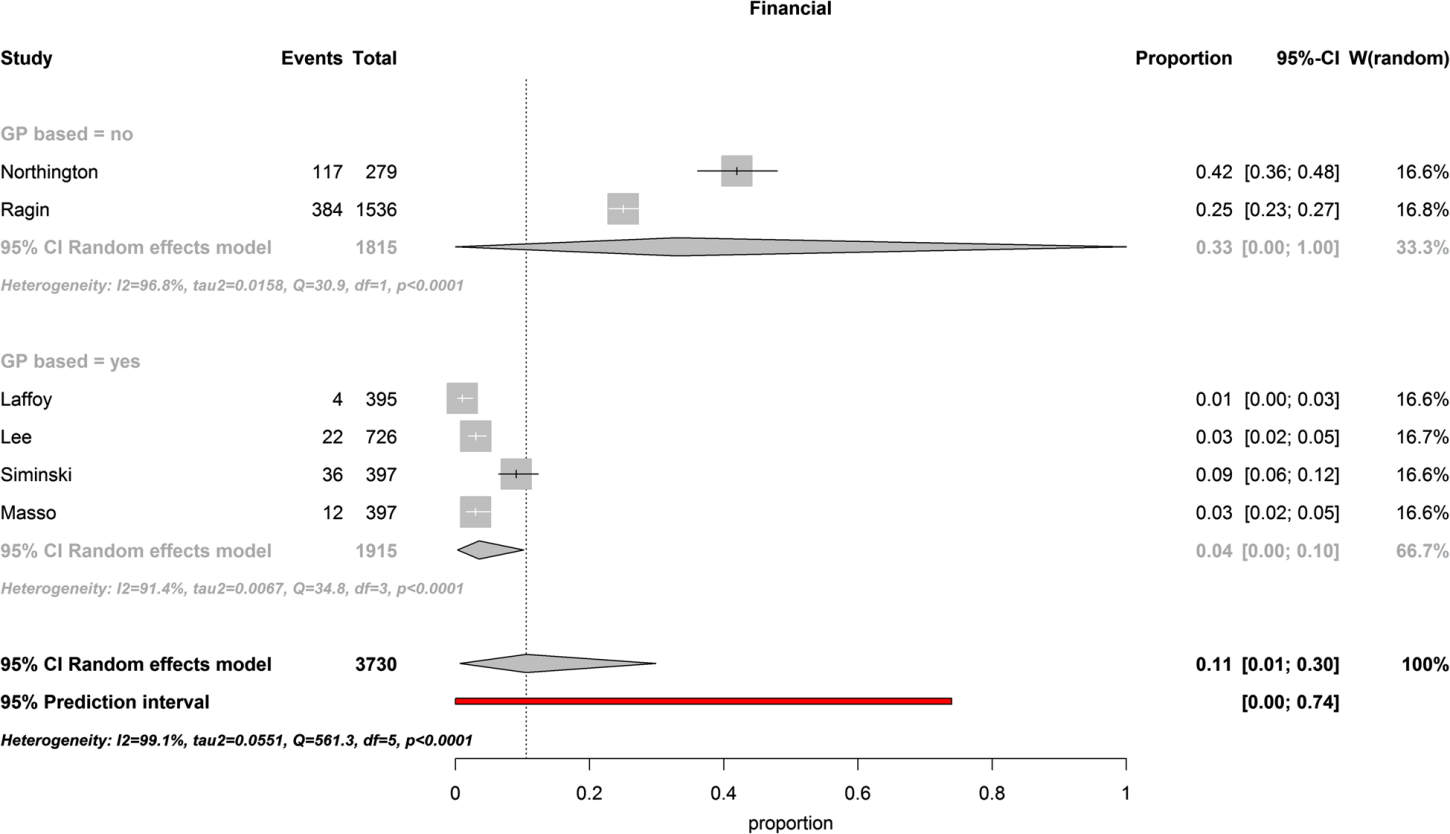
Self-referred patients visiting the ED out of financial motives in GP-based countries versus non-GP –based countries (USA). The two studies originating from the United States, reporting on financial considerations as a reason for self-referring to the ED, found significantly higher percentages of self-referred patients visiting the ED for this reason than studies from other continents did

Financial considerations were reported by 11% (PI 0-74%) overall. Studies from the USA reported 33% of patients visited the ED because of financial considerations [29, 31, 32], followed by 6% in Australia [37, 38]; 3% in Asia [35] and 1% in Europe [15] (*P* = 0.01). (Figure 2). Combining subgroups into non-GP-based countries (USA) versus GP-based-countries (remaining countries); we found 33% against 4% of patients citing financial considerations as reason for self-referral (*P* < 0.0001) (Fig. 2).

Studies including only adults [31, 32] found 33% (95% CI 0–100%) reporting on financial considerations, versus studies including patients of all ages [15, 35, 37, 38], with 4% (95% CI 0–10%), (*P* < 0.0001).

Lack of confidence in their GP was reported by 5% (PI 0-40%). Only studies from the UK [16, 24, 25] and the Netherlands [6, 13] reported on this reason for self-referral.

For none of the themes, the variation in the percentages could be explained by the use of multiple choice questions (with or without multiple possible answers) or the inclusion of only patients with a specific condition.

## Discussion

EDs are designed to provide emergency care and are not ideal locations for primary care, because there is no continuity of care, there is a risk for unnecessary testing and an ED-visit is more costly than a primary care visit [40]. This review shows that health concerns and the expectation to need further investigations are the most frequently reported motives to visit an ED without referral. Both motives reflect patients worried about their health, seeking urgent medical care. This is remarkable, because sixteen out of thirty of the selected studies only included patients with non-urgent problems. Patients may often be unable to judge the severity of their condition and may view non-urgent symptoms as urgent.

These two most common motives are difficult to address; there will always be differences between self-assessed and clinically assessed urgency and patients can only be expected to act on their own perceptions. Awareness programs that have been studied showed a limited effect. In one study, performed in the USA, people received a booklet with general information on when to visit an ED, but this did not show a significant effect on the number of ED-visits [41]. Education directed at specific conditions (ear pain in children, diabetes, asthma) and more intensive programs for geriatric or older, chronically ill patients have shown mixed results [42–47]. The effect of telephone consultation for patients to call for advice about their current health symptoms prior to seeking treatment at the ED also seems insufficient. In 1998, the UK introduced NHS Direct; a national nurse-led telephone advice service. Data suggested that this service reduced the number of calls to GP-cooperatives, but did not have a significant impact on the number of ED-visits [48]. Since 2014, NHS Direct has been replaced by NHS 111 with better integration with other health services. However, also NHS 111 has failed to reduce the number of ED-visits [49]. In the Netherlands, the implementation of ECAPs, a system where patients who unnecessarily visit the ED can be triaged to GPs, showed promising results in decreasing ED-utilization [50].

Health care systems are different between countries. The largest differences consist of how primary care is organized and the charges patients face when consulting a GP or ED. The results of this review should therefore be interpreted in the context of these health care systems.

### Europe

#### Health care system

Most European studies were performed in the UK and the Netherlands. These countries have similar health care systems, which heavily rely on primary care and most patients have a personal GP. During out-of-office hours patients can visit GP-cooperatives or walk-in clinics to get primary care. GPs are supposed to act as gatekeepers to secondary or specialist care, but patients can attend the ED without a referral if their condition, in their opinion, seems sufficiently urgent to them. In the Netherlands, people have a deductible excess charge of € 385 a year (in 2016); the first € 385 of medical bills, including the costs of an ED-visit, are charged to the patient. In contrast, emergency care is free of charge in the UK. GP-care is free of charge in both countries [51–53].

Despite the well-developed primary care systems, both countries have substantial numbers of self-referred ED-visits. Hospital Episode Statistics reported that in 2012–13, 64.1% of ED-visits (also including visits to minor injury units and walk-in centres) in England were self-referred [54]. In the Netherlands, 30% of ED-patients were self-referred in 2012 [8]. It has been shown that many of these patients visit the ED inappropriately [9, 52]. At the same time, ED crowding and ED waiting times are increasing, which underlines the importance of reducing the number of inappropriate self-referred patients [8, 55, 56].

#### Study findings

European studies found that patients reported visiting the ED because they expected that they needed laboratory or radiological investigations. Patients cannot get the same level of care with their GP and they visit an ED, when they expect that more advanced care will be necessary. A well-established primary care system does not change this.

Only studies from the UK and the Netherlands, reported a lack of confidence in their GP as a reason for self-referral to an ED, albeit with a low percentage. However, this is probably merely a reflection of the strong primary care network.

#### Practice implications

In the Netherlands, recent years an increasing number of EDs and GP-cooperatives are collaborating by creating Emergency Care Access Points (ECAPs) to reduce the number of self-referred ED-visits. During out-of-office hours, patients register at a conjoint desk, from where they are triaged to be seen by a GP or at the ED. This system shows promising results and is associated with an overall decrease in the number of ED-visits, almost disappearance of self-referred patients and a higher probability of hospital admission [50].

### USA

#### Health care system

The health care system of the USA, developed largely through the private sector, and combines high levels of funding with a low level of government involvement [57]. It has a small proportion of GPs and relies heavily on internal medicine and pediatrics for primary care [7]. In addition, the USA used to have a large proportion of uninsured or underinsured patients and patients often faced high cost sharing, including deductibles for primary care [57]. Because EDs are the only place where the poor could not be turned away, EDs were disproportionally used by low-income and uninsured patients who could not afford care in other settings [58]. In an attempt to deter inappropriate visits from EDs, several states implemented co-payments for non-emergency visits.

Recently, the health care system in the USA has undergone several changes, with the implementation of the Patient Protection and Affordable Care Act (PPACA) since 2010. With PPACA the percentage of uninsured patients is declining [59]. In addition, the funding for health centers was increased, which deliver preventive and primary health care to patients, regardless of their ability to pay. Between 2007–2015 these health centers have increased the number of patients served from 16 million, to 24 million annually [60].

Despite these measures, it seems that the number of ED-visits is still increasing: from 95 million in 1997, to 130 million in 2010 [61, 62]. In 2015, the American College of Emergency Physicians (ACEP) found that the majority of emergency physicians have noticed an increase in the volume of emergency patients since the requirement to have health coverage took effect in the PPACA in 2014 [63]. In addition, the number of EDs has decreased over the last years. Together, this leads to more overcrowded EDs [64].

#### Study findings

Studies from the USA reported significantly more frequently on issues with health-insurance and costs. This is to be expected, considering the charges patients faced when seeking medical care. However, all included studies were performed before the implementation of the PPACA, so it is not clear whether this affects the motivation of patients to visit the ED.

#### Practice implications

New research is necessary to see whether the motives for self-referral have changed since the PPACA was introduced.

### Australia

#### Health care system

Australia has a complex health care system, with public and private funders and providers; including public and private hospitals with EDs. Medicare, the tax-funded national health insurance scheme, offers patients free, self-referred access to the ED. GPs act as gatekeepers to the rest of the health care system, since patients need a GP-referral to consult a specialist [65].

It is estimated that the number of public ED-visits increased by 3.4% on average each year between 2010 and 2015. In 2014–15 there were about 7.4 million ED-consultations in public hospitals; 75% of patients who visited the ED had an arrival mode of ‘Other’; meaning they walked in or came by private or public transport, community transport or taxi. Ten percent were triaged as non-urgent [66].

#### Study findings

Studies from Australia found the highest percentage of patients visiting the ED out of health concerns and with the expectation to need investigations. There is no clear explanation for this finding.

#### Practice implications

Both motives are difficult to address.

### Overall

Studies have shown that a strong primary care network may help to reduce the number of self-referred patients in the ED, especially when patients have access to a GP for immediate care [67]. In our study, 13% of self-referred patients visited the ED because of the limited accessibility of primary care. So, better organization of primary care, with fast and easy access, might reduce the relatively small, but substantial number of patients self-referring to for this reason. Remarkably, we found no difference between continents in the percentage of the theme ‘accessibility of the GP’ was reported, despite the varying accessibility of primary care in the different healthcare systems. This might be because this theme reflects patients not getting a timely appointment with their GP in one country versus not having a personal GP in another country. Despite the well-established primary care in Europe and Australia, the number of non-urgent patients in EDs is substantial. This may be caused by the fact that the countries that have well established primary care systems also have well established healthcare insurance systems and historically have low thresholds for seeking medical consultation.

The results of this study show that health concerns are a major motivation for patients to self-refer to the ED, including for patients with non-urgent symptoms. This might be an important explanation for the limited effects of previous interventions; people who are worried about their health, will not be easily discouraged in seeking help at the ED. A solution in which a medical professional can triage self-referred patients to either a GP or the ED could relieve the patient of the burden of choosing the appropriate facility to present to, without discouraging patients to seek urgent medical care if needed. We believe the introduction of ECAPs may be that solution; the data on the effectiveness of ECAPs is promising, but is limited and subject to future research of our group.

#### Strengths and limitations

Strength of this study is that it reviews motives from self-referred patients worldwide, which provides data on what motives patients have to seek urgent medical care in EDs. These data can be used by policymakers to adjust healthcare systems in order to decrease self-referral associated costs. In addition, this study interprets the results of this review by taking into account the differences of healthcare systems in which the studies were performed.

This study only includes studies in Dutch and English and might therefore have missed some relevant articles.

Seven articles used multiple choice questions, with the option of selecting multiple answers [12, 13, 15, 16, 33, 37, 39]. Unfortunately, it is not clear from these articles how many patients selected multiple answers. This makes it impossible to assess what reasons were most important for these patients in self-referring to the ED.

This review could not explore whether motives for appropriate and inappropriate visits differ, because the included studies did not report on the appropriateness of ED-visits.

Large variations in reported percentages of reasons for self-referral between studies were found, reflected by wide prediction intervals and high levels of heterogeneity. Subgroup analyses were performed in order to analyze whether this could be explained by different healthcare systems or study methods, but not all heterogeneity could be explained. It is plausible that other, unknown factors that are not reported in the original manuscripts influence the reported percentages and the inability to explain reporting heterogeneity might therefore be.

## Conclusion

Reasons for self-referral to EDs differ slightly with different healthcare systems. Worldwide, the most important reasons to self-refer to an ED are health concerns and additional investigations. Financial considerations mainly play a role in the United States.

## Appendix 1

**Table 4:** Included studies with description of method, number of included patients and results

Article	Method	Number of patients	Reasons for self-referral
Europe			
Mestitz [28] United Kingdom 1957	Questions asked by casualty medical officer	975 (770 self-referred patients) Only adults? Only medical patients	The commonest reply was that it was more convenient to come to the hospital than go to the surgery. A few were genuinely surprised when I told them that the proper course was to consult their own practitioner first. This leaves those patients -quite a considerable number- who, without admitting that they did not trust their own doctor, indicated that they thought that better treatment would be meted out to them in hospital. The two chief factors in this group were the feeling that x-ray examinations were more readily ordered in hospital, and that a hospital doctor would carry out a more thorough examination. Mothers who brought their children often gave this last answer.
Wilkinson et al. [24] United Kingdom 1977	Interviews, using questionnaires	546 (213 first attenders registered with a GP near enough to visit and who had neither come by ambulance nor been sent in to see a doctor immediately by the casualty receptionists for emergency treatment) All ages Exclusion: emergency admissions, suffering from alcohol or addictive drugs	14% Needs hospital treatment 17% Considered urgent 1% Previous patient at hospital 14% Hospital more convenient 2% GP too far away 4% Did not want to lose work time 14% GP away or not available 5% Did not wish to wait for GP appointment 11% Sent by someone (usually employer) 2% Dissatisfied with GP 9% Other reasons 7% Don’t know
Myers et al. [26] United Kingdom 1982	Question asked	150 Only adults? Exclusion: collapse, abdominal and chest pains, acute gynecological problems, overdose and major medical problems	Problem thought to need hospital tests or treatment 71 (47%) Could not wait for GP appointment 32 (21%) Referred to hospital by employer, nurse, etc. 13 (9%) Miscellaneous (e.g. hospital nearer, is open all night, dislike of GP, don’t know) 19 (13%) Requesting second opinion 7 (5%) Happened to be in hospital anyway 3 (2%) Does not have GP 5 (3%)
Singh [21] United Kingdom 1988	Interviews, using semi-structured questionnaire	217 All ages	Eighty nine patients cited urgency as afactor in their decision to bypass the general practitioner and go direct to the casualty department. Fifty three patients thought that they would need an x rayexamination and gave this as the reason for self-referral. Thirty nine patients thought that their doctor was not available after surgery hours and 16 that it would be quicker going to the casualty department. Other responses included advice from friends and relatives (15 patients) and being out of the practice area at the time of the emergency (14). Twelve patients specifically cited not wanting to bother their doctor as their reason for attendance.
O’Halloran et al. [16] United Kingdom 1989	Questionnaires Multiple choice, multiple answers possible	145 (124 self-referred patients) Age: 18 months to 16 years. Visited with acute asthma, at least one more visit in the preceding 12 months.	40% GP said to go to the hospital if child bad / have always been sent to AED so now go straight here 30% Quicker to go to AED than to wait for GP or locum to visit 29% Nebuliser only thing that helps 21% Little confidence in GP 11% Better facilities for treatment in hospital 10% No point calling GP because he can’t do anything parents haven’t done already 7% Feel safer in hospital 6% Told to come by hospital staff 4% Have nebulizer at home and need to go if that fails 9% Other reasons
Stewart et al. [18] United Kingdom 1989	Questionnaires	853 (585 self-referred patients) Children	20.9% Anticipated referral 16.2% Better treatment at hospital 11.5% Always come to hospital 6.5% Wanted second opinion 10.3% Hospital more convenient 9.6% Too long wait for family doctor 5.6% Too difficult to contact family doctor 4.3% Hospital always open 5.9% Did not want deputizing bureau doctor 7.5% Patient attending for this condition 0.7% Patient attending hospital for other condition 1.2% Missing information
Thomson et al. [19] United Kingdom 1995	Questionnaires Multiple choice	245 (147 self-referred patients) Only adults? Non-emergency	15% Easier geographical access 24% Convenience related to timing 59% GP’s perceived inability to treat disorder 3% Other
Ward et al. [25] United Kingdom 1996	Question asked by treating physician	970 All ages Primary care problem	Question answered by 339 patients: Problem not appropriate for GP 92 (27,1%) Not convenient to see GP 76 (22,4%) Advised by health professional 39 (11,5%) (health professional not specified, not the GP) Second opinion 33 (9,7%) Did not try to see GP 33 (9,7%) Appointment not available with GP 25 (7,4%) Unable to contact GP 21 (6,2%) Dissatisfied with GP 15 (4,4%) Other 5 (1,5%)
Laffoy et al. [15] Ireland 1997	Interviewer-administered questionnaires Multiple choice, multiple answers possible	557 (395 self-referred patients) All ages	35.4% Thought I needed immediate attention 18.2% Thought I needed an X-ray 13.7% Hospital is convenient 7.6% Thought GP would refer me anyway 7.1% I prefer hospital for this condition 5.6% I’m under hospital care already 0.8% Hospital cheaper than GP 0.3% GP told me to go to A&E 14.4% Other
Shipman et al. [23] United Kingdom 1997	Telephone interviews, semi-structured	82 All ages	When the patient had not attempted to contact their GP or deputizing service prior to attending A&E, reasons included seeing A&E as the appropriate service for a particular problem, in particular when the problem started suddenly and A&E was seen as having the most appropriate diagnostic service. For some A&E attendees, decision making appeared to be have been less related to perceptions of appropriateness than to service availability. In some cases it was assumed that there was no out-of-hours general medical service available. For other respondents, A&E was seen as the speediest option for seeing a doctor.
Rieffe et al. [17] the Netherlands 1999	Questionnaires, Likert scale	430 Only adults? ‘Could have been seen by a GP’ Exclusion: too confused or in too much pain to complete questionnaire	Profiles of two major patient groups could be identified. One group comprised patients with a high socio-economic status living in suburbs, whose motives for visiting the ED are mainly of a financial nature. Patients in the second group mainly lived in the inner city and preferred the expertise and facilities provided by the ED.
Jaarsma-van Leeuwen et al. [5] the Netherlands 2000	Questionnaires Multiple choice	1068 All ages. Only surgical patients	6.1% own GP not in area 2.6% no trust in GP 47.7% not thought of GP 11.4% did not want to wait for an appointment with a GP 28.3% wanted specialist care (eg radiologic investigations) 3.9% otherwise (treated in the hospital, working in the hospital, no personal GP, could not reach GP)
Rajpar et al. [22] United Kingdom 2000	Interviews, using semi-structured questionnaire Multiple choice	54 All ages ‘Primary care problem’	50% ‘GP was closed’ 3.7% tried to contact GP 22.2% Perceived severity of problem 11.1% Did not want to disturb GP 7.4% Wanted second opinion 5.6% Perceive wait at A&E shorter than at GP cooperative 3.7% Perceived that facilities and investigations better at A&E
Coleman et al. [12] United Kingdom 2001	Questionnaires Multiple choice, multiple answers possible	255 Adults Patients with ‘low priority for treatment’	38% Availability of other services 62% Awareness of other services 11% Patient preference 70% Positive experiences of A&E 56% Processes and patient’s time 24% Convenience of access 76% Perceptions of seriousness 38% Seeking assurance 43% Other directed 68% Seeking particular services (all subdivided into smaller categories)
Norredam et al. [27] Denmark 2007	Questionnaire Multiple choice	2746 Age > 14 years Ambulatory patients	13% I could not get in contact with a GP 63% The ER is most relevant to my need (24% I was referred by a primary caregiver)
Moll van Charante et al. [3] the Netherlands 2008	Postal questionnaires Multiple choice	808 (224 self-referred patients) All ages	36% Further research (eg X-rays) was necessary 30% The doctor in the AED is best qualified for this problem 16% The AED is better accessible than the GP cooperative 5% It was related to a recent hospital contact or procedure 4% I didn’t want to disturb the GP / no GP available 5% Other 4% Missing
Mc Guigan et al. [14] United Kingdom 2010	Interviews by telephone, semi-structured	196 Age > 16 years Exclusion: patients who were seriously ill or otherwise vulnerable	48% Perceived appropriateness of condition 35% After taking advice from others 3% Anticipation of referral by GP 6% Accessibility of ED 5% Unavailability of GP 1% Other
van der Linden et al. [20] the Netherlands 2014	Open question by triage nurse	3028 All ages	1751 self-referred patients answered the question (58%): 34% Accessibility and convenience 27% Perceived medical necessity (Less often, no percentages given: Not thought about GP, Not having a regular GP, Familiarity, Dissatisfaction with GP, Referral by non-professionals)
de Valk et al. [13] the Netherlands 2014	Questionnaires Multiple choice, multiple answers possible	436 Age > 18 years Exclusion: patients who were unable to fill out the questionnaire	28% Patients’ assumption that medical care was needed that a GP cannot provide (eg. X-ray, blood tests) 17% Patient was already under specialist care at the study hospital 16% Patient could get help earlier at the ED 11% The ED was nearby 11% Patient was not registered with a GP 7% Patient could not reach the GP/GP-cooperative 5% The location of the GP-cooperative was unknown 4% Previous negative experience with the GP/GP-cooperative 3% Patient had no faith/trust in the GP 3% On the advice of others 2% Patient perceived the complaint was urgent
North America			
Hunt et al. [33] USA 1996	Questionnaires Multiple choice, multiple answers possible	1547 All ages	Columbia Grand Strand Regional Medical Center (tourist community) (n = 548): 126 23.0% “I’m from out of town and just looked for the nearest emergency room.” 119 21.7% “Don’t have a doctor/clinic that regularly takes care of me.” 110 20.1% “Don’t have to make an appointment at the emergency room.” 86 15.7% “Better medical care here than other places.” 80 14.6% “My problem is bigger than my regular doctor/clinic could take care of.” 66 12.0% “My doctor/clinic told me to come to the emergency department when the office is closed.” Pitt County Memorial Hospital (training program): n = 990 responses 154 15.6% “Don’t have a doctor/clinic that regularly takes care of me.” 142 14.3% “Better medical care than other places.” 126 12.7% “Don’t have to make an appointment at the emergency room.” 109 11.0% “My doctor/clinic told me to come to the emergency department when the office is closed.” 75 7.6% “My doctor couldn’t see me soon enough.” 70 7.1% “My problem is bigger than my regular/clinic could take care of.”
Koziol-McLain et al. [34] USA 2000	Interviews, qualitative methodology	30 Age > 18 years (despite this inclusion criterion 1 patient of 17 years was included) Non-urgent	5 themes were found - Toughing it out - Symptoms overwhelming self-care measures - Calling a friend - Nowhere else to go - Convenience
Northington et al. [31] USA 2004	Questionnaires + brief interview	279 Age > 18 years Non-urgent	76.1% Better care 73.6% Urgency 68.6% Immediacy 41.9% Payment flexibility 39.7% Expediency
Howard et al. [30] USA 2005	Interviews, qualitative methodology	31 Age 18–50 years Non-urgent complaints	Three major themes: - They were unable to obtain an appointment with a PCP - They were referred by the staff (not the doctor) in PCP’s offices to be evaluated in the ED - It took less of their time to be seen in the ED than it did to contact their PCP, only to be told to go to the ED
Ragin et al. [32] USA 2005	Interviews, questionnaires. Likert scale	1536 Age > 18 years Not cognitively or medically impaired	Medical necessity was the most frequently cited reason (95.0%), followed by convenience (86.5%), ED preference (88.7%), affordability (25.2%), and limitations of insurance (14.9%).
Grant et al. [29] USA 2010	Interviews, qualitative methodology	112 Children Non-emergency	The majority of participants cited some aspect of clinic or pediatric office operations as the principal reason for coming to the ED. Other problems cited included clinic capacity, inconvenient appointment times and long waits for appointments. Several caregivers said they preferred to obtain care at the ED because they could be seen on a walk-in basis. Some parents reported problems getting their health coverage transferred after moving, and knew they could be seen without insurance at the ED. Also a frequently cited reason for the ED visit was the need for follow-up care.
Asia			
Shah et al. [36] Kuwait 1996	Questionnaires, open ended question	1146 Only adults?	27.8% Hospital better or clinic worse/medicine not available 59.8% Accessibility/availability of ER 11.0% Have ‘wasta’ (connection or social intermediary) 14.0% Worker in hospital 7.5% Clinic closed or not available or do not know clinic timings 13.2% Hospitals close by or convenient 12.1% Have file, appointment, regular patient 2.0% Refused by PHC 10.7% Condition urgent 1.6% Other
Lee et al. [35] Hong Kong 2000	Telephone interviews, using questionnaires	2410 (726 patients with conditions that could be treated by GPs) All ages	For those patients who attended A&E with conditions that could be treated by GPs, main reasons were: 43.8% Perceived emergency status of their disease 28.9% Feeling sick on public holidays or at night 12.4% Living in close proximity to the hospitals 11.4% Availability of proper diagnosis and efficient service at the time of day it was needed 3.4% Low cost Other factors which also demonstrated statistical significance were the desperate need for help, the feeling that the situations could best be handled in the A&E facility, and the fact that patients had been sent to the department from school or from their workplace
Australia			
Masso et al. [38] Australia 2007	Questionnaire, Likert scale	397 All ages ‘Primary care patients’, category 4 or 5 of the Australasian Triage Scale	67.3% My health problem required immediate attention 38.2% My health problem was too serious or complex to see a GP 15.4% I feel the medical treatment is better at the ED 5.7% I wanted a second opinion 1.6% Id did not want my GP to know about this health problem 3.4% I usually prefer to talk a doctor a don’t know about my health problems 51.3% I am able to see the doctor and have any tests or X-rays all done at the same place 7.6% I am not able to get in as a patients at GP surgery as the books are closed 12.6% I am not happy with the time I have to wait to get to an appointment with a GP 4.2% I do not like making appointments 8.4% It is easier for me to go to the ED 2.9% There is no charge to see a doctor at the ED 3.4% There is no charge for X-rays or medicine at the ED 0.5% I wanted to see a female doctor 0.8% Doctor or interpreter who speaks my language 1.3% Aboriginal health staff 1.3% Prefer ED environment 2.6% Traditional use by family
Siminski et al. [37] Australia 2008	Questionnaires Multiple choice, multiple answers possible	400 All ages Low urgency /acuity	Patients could choose multiple answers from 18 options. The most striking finding was the consistency of the most prevalently selected reasons across all age groups. Self-assessed urgency, access to diagnostics and self-assessed complexity were selected most often. 80% Problem too urgent 53% Problem too serious/complex 34% Medical treatment better at ED 14% Second opinion 2% Did not want the GP to know 6% Prefer doctor I don’t know 74% See doctor and have tests/x-rays done in same place 16% Not able to see GP as books are closed 24% Not happy with GP waiting time 12% Do not like making appointments 21% Easier to get to the ED 9% No charge to see a doctor 10% No charge for X-rays or medicine 2% Female doctor 2% Doctor or interpreter who speaks my language 2% Aboriginal health staff 5% Prefer ED environment 9% Traditional use by family
Others			
Rassin et al. [39] Isreal 2005	Questionnaire Multiple choice, multiple answers possible	73 Age > 18 years Home-discharged	62.86% of the participants reported that they had decided to go to the ER since the quality of treatment there was higher compared to the community local clinic. 47.17% indicated that the geographical proximity of the ER to their residence had led them to turn to it for medical treatment. This reason was especially prominent (66.67%) among the 70 and older age group. 68.57% indicated that they had decided to visit the ER following a recommendation of a relative. Factors analysis using linear regression, conducted to examine what had most influenced the decision to go to ER, showed that relatives’ recommendation had an overwhelming affect (*b* = 0.333, *P* = 0.012).

## Appendix 2

**Table 5:** Reasons for self-referral categorized in themes

Study		Country	*N* (total)	Percentages per theme						
Ref. nr.	Author			Health concerns	Expecting investigations	Convenience of the ED	Lesser accessibility of primary care	No confidence in GP/primary care	Advice from others	Financial considerations
[13]	De Valk	The Netherlands	436	2	28	16	7	4	3	
[33]	Hunt2	USA	990	7		13	11			
[12]	Coleman	UK	255	11	48	13	5		43	
[16]	O’Halloran	UK	124	11		30		21	6	
[36]	Shah	Kuwait	1146	11		13	8			
[33]	Hunt	USA	548	15		20	12			
[18]	Stewart	UK	585	16		10	10			
[24]	Wilkinson	UK	213	17		14	14	2	11	
[22]	Rajpar	UK	54	22	4	6	50			
[20]	Van der Linden	The Netherlands	1751	27		34				
[25]	Ward	UK	339	27			22	4	12	
[3]	Moll van Charante	The Netherlands	224	30	36	16	4			
[15]	Laffoy	Ireland	395	35	18	14				1
[21]	Singh	UK	217	41	24	7	18		7	
[35]	Lee	Hong Kong	726	44		12			-	3
[14]	McGuigan	UK	196	48		6	5		35	
[27]	Norredam	Denmark	2746	63			13			
[39]	Rassin	Israel	73	63		47			69	
[38]	Masso	Australia	397	67	51	8	8			3
[31]	Northington	USA	279	74						42
[37]	Siminski	Australia	400	80	74	21	16			9
[32]	Ragin	USA	1536	95		87				25
[17]	Rieffe	The Netherlands	430	-						-
[29]	Grant	USA	112	-		-	-			-
[5]	Jaarsma van Leeuwen	The Netherlands	1068		28		11	3		
[26]	Myers	UK	150		47	2	21		9	
[23]	Shipman	UK	82		-	-	-			
[28]	Mestitz	UK	770		-	-				
[19]	Thomson	UK	147			24				
[30]	Howard	USA	31			-	-		-	
[34]	Koziol-McLain	USA	30			-			-	

- = Reported, but no percentages given/qualitative study

## Abbreviations

CI: Confidence interval
ECAP: Emergency care access point
ED: Emergency Department
GP: General practitioner
PI: Prediction interval
UK: United Kingdom
USA: United States of America
